# Estimated Osmolality by Measured Conductivity in 24 h Urine Renal Stone Patients: A Useful Tool for Monitoring Dietary Sodium and Protein Excess

**DOI:** 10.3390/jcm14196898

**Published:** 2025-09-29

**Authors:** Louange Luemba Sita, Pitchouna Ingole Mboliasa, Ernest Kiswaya Sumaili, Vincent Frochot, Remi Chieze, Emmanuel Letavernier, Jérémie Muwonga Masidi, Mireille Nganga Nkanga, Michel Daudon, Jean Philippe Haymann

**Affiliations:** 1 Renal Unit and Medical Biology Department BP 818 Kin XI, Kinshasa University Hospital (KUH), Université de Kinshasa, Avenue de l’Université, Kinshasa 999069, Democratic Republic of the Congo; mboliasa@yahoo.fr (P.I.M.); sumaili.ernest@upc.ac.cd (E.K.S.); pmuwonga@hotmail.com (J.M.M.); mnganga2002@yahoo.fr (M.N.N.); 2 Faculty of Medicine BP 4745 Kin II, Université Protestante au Congo, Croisement des Avenues Liberation et Triomphal, Kinshasa P.O. Box 4745, Democratic Republic of the Congo; 3 Assistance Publique Hôpitaux de Paris (APHP), Sorbonne Université, GRC n°20, INSERM UMRS 1155, Hôpital Tenon 4 Rue de la Chine, 75020 Paris, France; vincent.frochot@aphp.fr (V.F.); remi.chieze@aphp.fr (R.C.); emmanuel.letavernier@aphp.fr (E.L.); daudonmichel24@gmail.com (M.D.); jean-philippe.haymann@aphp.fr (J.P.H.)

**Keywords:** urine conductivity, urine osmolality, stone patients, 24 h diuresis, sodium intake, protein intake

## Abstract

**Background/Objectives:** Monitoring of 24 h urine analysis is currently used to assess diet-related stone risk factors due in most cases to low hydration and high osmole intake accounting for urine supersaturation. The aim of our study is to test whether urine conductivity could be a relevant surrogate marker of urine osmolality and a useful tool for monitoring salt and protein diets in primary care centers. **Methods:** 113 patients with kidney stone history referred for a routine evaluation of fasting and 24 h urine samples were included. Biochemical analysis of urine was performed, including measured osmolality (mUosm) and conductivity. **Results:** Among our population, 45% of patients have a low diuresis (high-risk group of stone recurrence) below the target of 2 L/day, with lower daily mUOsm and conductivity outflow compared to the low-risk patient group > 2 L/day (718 versus 852 mosm/Day, *p* < 0.0001, and 13,730 versus 17,890 mS/cm/day, *p* < 0.0001, respectively). Conversely to urine sodium and urea concentration, daily sodium and protein intake estimated by natriuresis and urea excretion are significantly lower in the high-risk group (*p* = 0.01 and <0.0001, respectively). In 24 h urine samples, osmolality and conductivity were strongly associated with diuresis. Moreover, a strong association between urinary osmolality and urine conductivity enables an estimated osmolality (eUosm) according to the following equation: eUosm = −41.656 + 0.057 × conductivity (r^2^ = 0.93; *p* < 0.001) with a 95% limit of agreement (LoA) ranging from −7.2% to +7.3%. An eUosm threshold value < 900 mOsm/day is independently associated with sodium and protein intake targets (odd ratio: 19.2 and 6.4-fold, respectively, *p* < 0.0001 and 0.01). **Conclusions:** 24 h urine measured conductivity appears to be a reliable, easy-to-use tool for the screening and monitoring of diet-related stone patients in primary care centers.

## 1. Introduction

Urinary lithiasis, a disease characterized by stone formation in the urinary system, is a major public health problem. The causes are diverse, including drugs, dietary and urological abnormalities, hormonal disorders, intestinal malabsorption, and infection, among others.

Stone disease may induce kidney, bone, and infectious complications, making prevention essential [1]. It also represents a heavy economic burden for healthcare systems, with a significant workday loss estimated at EUR 300 million per year in France [2].

Currently, in industrialized nations, it affects approximately 10% of the adult population. Recent epidemiological studies confirm a marked upward trend in its prevalence over the past three decades. This increase is primarily linked to changes in socioeconomic status, lifestyles, and dietary habits. For example, in Germany, a nearly 20% increase in stone cases was reported over the past 20 years [3].

Urinary lithiasis is a highly recurrent disease: the prevalence of stone recurrence after a first stone episode reaches about 50% within five years of follow-up, and in some cases may lead to end-stage renal failure [4]. Efficient monitoring in order to assess stone risk factors and prevent recurrence and renal failure and, thus, appears indeed as a relevant cost-effective health investment [5,6].

Urine dilution, in all cases, appears as a key factor to prevent stone recurrence. Monitoring of 24 h urine, fasting urine, or spot urine collections (depending on medical teams’ habits) is performed on a daily basis in order to assess stone risk factors and initiate appropriate care [7]. Of note, aside from urine dilution, 24 h urine analysis alone also provides additional valuable diet information, namely water, sodium, and protein daily intake, a useful tool for patient diet monitoring. In order to assess more precisely stone risk factors, i.e., urine supersaturation for crystal formation, several indexes such as the Tiselius index, EQUILs, RSS, RRFA, or BRI were developed [8].

These different indices quantify the risk of stone formation based on the analysis of the concentration of certain urinary parameters. Indeed, the Tiselius index and the Bonn Risk Index assess the risk of calcium oxalate stone formation using different methods; the first takes into account urinary parameters such as calcium, oxalate, citrate, magnesium, phosphate, pH, and urine volume to determine urinary supersaturation, while the second is based on a crystallization test carried out on urine samples, taking into account the ratio between free ionized calcium and ammonium oxalate [8]. RRFA essentially assesses the risk of uric acid stone formation by adding uric acid as one of the urinary parameters to be considered, and RSS requires up to 14 urinary parameters to assess the risk of stone formation of different types [9].

Despite relevant prediction for stone recurrence [9], index assessment requires tedious calculation, limiting their daily practice use, whereas urine osmolality, specific gravity, or conductivity measurements may provide straightforward information about urine dilution. As a matter of fact, urine osmolality measured by delta cryoscopy, expressed in mosm/kg, is considered a gold standard to quantify precisely renal concentration or dilution performance but also body hydration [10,11]. However, osmometers are not easily available, especially in primary care centers, explaining the widespread use of specific gravity instead, which may also be used at bedside. Urine gravity dipstick unfortunately includes some bias related to high or low pH value, which may under- or overestimate true gravity using a densitometer but also show discrepancy with osmolality measurements in some patients having an unusually high ratio of high molecular weight solutes [12]. Interestingly, urine conductivity measurement, which is independent of solute molecular weights, may be an interesting tool to assess urine dilution, as easy-to-use portable conductivity meters are available on the market with good accuracy [13,14]. Few reports indeed show an association between measured urine osmolality and conductivity, in the general population, stone patients, and chronic kidney disease patients, despite conflicting results about accuracy [5,9,15].

The aim of our study is to test whether measured urine conductivity in 24 h and/or fasting urine samples could be a relevant surrogate marker of measured urine osmolality and a potentially useful tool for the screening and monitoring of dietary targets in stone patients.

## 2. Materials and Methods

We analyzed both 24 h and fasting urine of 113 renal stone patients referred to our center in Tenon Hospital between January and July 2022 with a complete routine metabolic workup. All patients gave informed written consent to have their data collected anonymously for biomedical research. Biologic evaluation included biochemical analysis of 24 h urine and fasting urine samples, noteworthy urine osmolality and conductivity, pH, sodium, potassium, calcium, phosphate, urate, magnesium, oxalate, and citrate.

Urine osmolality was measured by freezing-point depression, and urine conductivity was conducted using a pH and conductivity meter T50 (Mettler Toledo SAS, Viroflay, France) which measures urinary conductivity using an integrated temperature-controlled microprocessor.

Urine osmolality was measured using an osmometer (Osmo1, Radiometer, Neuilly-Plaisance, France), which measures the decrease in the freezing temperature of a solution relative to that of a pure solvent. This decrease is proportional to the solute concentration.

To measure urinary osmolality, we proceeded as follows: After calibrating the equipment, 20 microliters of the fasting or 24 h urine sample, previously prepared either by direct mixing or by centrifugation if cloudy, was introduced into the measuring cell using an appropriate pipette. The device performs the measurement and displays the value in mosm/kg H_2_O on the screen.

To measure urinary conductivity, we first selected the conductivity mode, then the equipment was calibrated while carefully rinsing the conductivity probe with distilled water. Finally, we started the analyses by immersing the probe in the urine sample and then waited for the results to be displayed on the screen, expressed in µS/cm.

Urine sodium, potassium, and pH were analyzed using a specific electrode for each parameter in a urine biochemistry analyzer (ABL 815, Radiometer, France).

Calcium, magnesium, and phosphate using a standard colorimetric method, and creatinine by enzymatic method using the INDIKO analyzer (Thermo scientific, Paris, France).

For validation of the results, it was imperative to perform quality control for each parameter used. Therefore, there was a normal control and a pathological control (high and low). The quality control results were within acceptable ranges.

Clinical and biological data were collected from the department of physiology database (data collection was approved by the “Commission Nationale de l’Informatique et des Libertés” according to French legislation, n◦2065902v0).

## 3. Statistical Analysis

Qualitative and quantitative data were expressed as percentage or median, respectively. The comparison of quantitative and qualitative data was carried out according to the Mann–Whitney and Chi-squared test, respectively. Univariate associations were tested according to the Spearman non-parametric test. In order to develop an estimated urine osmolality (eUosm) equation, we used a linear regression model to assess the relationship between urine conductivity and mUosm. To evaluate the performance of the equation, we defined mean bias as the mean difference between eUosm and mUosm; accuracy was estimated by the percentage of patients with estimated osmolality slopes; within 20% and 30% of their measured osmolality slopes, and the 95% limits of agreement (LoAs) were calculated as mean bias +/−2 SD around the bias. Visual representations of the agreements were provided as Bland–Altman plots [16]. A *p*-value strictly less than 0.05 was considered statistically significant. All statistical analyses were carried out using Stat view 5.0 software.

## 4. Results

Among 113 renal stone patients, 54% of patients have a diuresis above the target of 2 L/day with a median serum creatinine of 85 versus 86 µmol/L in the group with a diuresis below 2 L/day (*p* = 0.94). Dietary sodium and protein excess (assessed, respectively, by a natriuresis > 150 mmol/day and urea > 400 mmol/day) is encountered in 33% and 24% of cases. As shown in Table 1, a comparison between low- versus high-risk patients (i.e., patients with a diuresis > or >2 L/day) shows that daily osmole outputs were higher in low-risk patients (852 versus 718 mOsm/day, respectively, *p* < 0.0001). Accordingly, sodium and urea outputs were also higher (mean values of 144 versus 119 mmol/day and 382 versus 283 mmol/day, respectively), with diet sodium and protein excess detected, respectively, in 44% versus 22% and 42% versus 8% of cases, respectively (*p* = 0.02 and *p* < 0.0001). However, urine osmolality and conductivity are significantly lower in high-risk patients due to dilution compared to low-risk patients (median value of 331 versus 475 mosm/kg and 6846 versus 9092 mS/cm, respectively). Of note, whereas fasting urine conductivity is significantly higher in low-risk patients with a median value of 11209 versus 9621 µS/cm (*p* = 0.02), fasting urine osmolality is surprisingly similar between the two groups (Table 1).

As shown, oxaluria output is increased in high-drinker group, whereas calcium, phosphate, urate, magnesium, and citrate concentrations are similar between the two groups.

**Figure 1:**
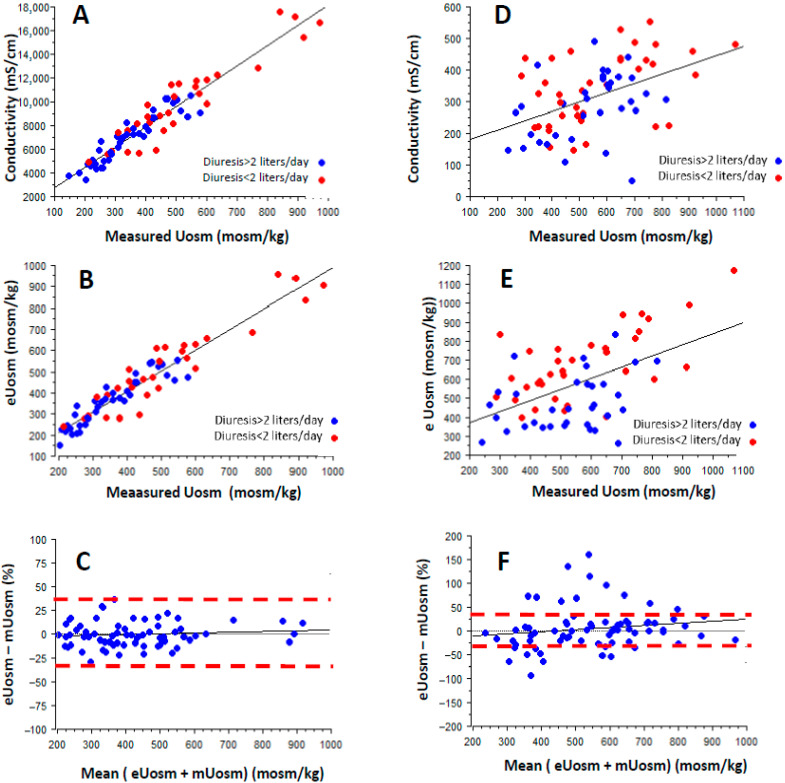
Association between measured conductivity and measured osmolality in 24 h urine collection (**A**) or fasting urine (**D**). Association between eUosm and mUosm in 24 h urine collection (**B**) or fasting urine (**E**). Bland and Altman representation of eUosm versus mUosm in 24 h urine collection (**C**) or fasting urine sample (**F**).

Table 2 shows significant associations between 24 h urine conductivity or osmolality and sodium and urea concentrations but also most urine parameters, including stone risk factors such as diuresis (Figure S1), calciuria, phosphaturia, uraturia, and pH. As shown Table 1 and Figure 1A there is a strong linear association between measured urine osmolality (expressed in mosm/Kg) and urine conductivity expressed in µS/cm, which enables an estimation of urine osmolality (eUosm) according to the following equation: eUosm = −41.656 + 0.057 × conductivity (r^2^ = 0,93; *p* < 0.001). A table of correspondence between 24 h urine conductivity and measured urine osmolality is provided in Table 3. According to this equation, urine conductivity appears as a good surrogate marker of estimated 24 h Uosm (eUosm) (Figure 1B,C) with a 95% limit of agreements (LoA) ranging from −7.2% to +7.3% and a P20% and P30% accuracy of 87.3% and 100%, respectively. Thus, from 24 h urine collection, eUosm allows us to estimate daily osmoles output of 712 and 940 mosm/kg/day in low- and high-risk groups, values close to mUosm of 718 and 852 mosm/kg/day. By contrast, in fasting urine, conductivity association with osmolality is weaker (r^2^ = 0.20, *p* < 0.0001) (Figure 1D–F) with a P20% and P30% accuracy of 53% and 82%, respectively (LoA ranging from −18% to +23%). Table 4 shows that an eUosm threshold below 900 mosm/Day is independently associated with no excess sodium and protein intake (odd ratio: 19.2- and 6.4-fold, respectively). Of note, a 24 h eUosm threshold < 350 mOsm/kg is independently associated with a diuresis sodium and protein intake within required targets (odd ratio: 15.2, 16.5, and 7.3, respectively, as shown in Table 4B) and increased the likelihood to meet all three criteria together by 15.7-fold (15.7 [4.7–53.0], *p* < 0.0001).

## 5. Discussion

Our data show that eUosm-derived equation from measured conductivity is a relevant surrogate marker for urine osmolality in 24 h urine collection and a potentially useful tool for diet screening in renal stone patients. Indeed, we report a strong association between 24 h urine osmolality and conductivity with a *p* 30% value of 100%, thus demonstrating that eUosm was indeed an accurate and robust surrogate marker of urine osmolality. The provided conversion table (Table 3) enables an easy-to-use tool to estimate urine osmolality, as shown in Figure 1B. Of interest, the estimated osmolality using the measured conductivity equation gives a better accuracy than the estimated urine osmolality using the usual Equation 2 × ([Na] + [K]) + [urea], as assessed by 95% limit of agreements (LoA) ranging from −7.2% to +7.3% and −34% to +23%, respectively.

Conversely to our 24 h urine data, in non-selected patients first morning urine specimens [14] or renal stone patients spot urine samples [10,17,18], despite urine osmolality and conductivity association, the sole use of conductivity was found to be misleading in the estimation of osmolality [19]. Indeed, Oyaert et al. reported that among 270 samples, 106 were outside the allowable range, with LoA ranging from −70.50% to 55.06% (i.e., −295 mOsm/kg to +238 mOsm/kg) [18]. These findings are somewhat in agreement with our fasting urine data showing a significant association between osmolality and conductivity but a poor P30% accuracy of only 82% (LoA ranging from −18% and +23%).

Differences between 24 h urine and fasting or spot urine results raise the issue of identifying bias at play. Actually, conductivity depends on negatively and positively charged molecules and does not take into account uncharged molecules such as urea [20], whereas measured osmolality depends on the number of solutes, either charged or neutral. Thus, urea alone must explain our conflicting results. Indeed, 24 h urea excretion is the witness of the patient’s protein diet and thus tightly linked with sodium, potassium, and ammonium salt intake, whereas the fasting urine concentration of urea depends primarily upon the duration of water restriction, renal concentration performance altogether with the protein diet. Therefore, eUosm appears as an accurate surrogate marker for osmolality in 24 h urine collection, providing also valuable information about the patient’s diet and water intake.

Indeed, as shown in Table 1, urine conductivity was lower in patients with a diuresis > 2 L/day compared to diuresis < 2 L/day, with measured osmolality values of 331 versus 475 mosm/kg, respectively, and remarkably close to estimated osmolality (363 and 496 mosm/kg, respectively). In accordance with previous reports, a diuresis > 2 L/day (i.e., a value below 7000 mS/cm corresponding to eUosm around 350 mosm/kg) appears as a relevant target to decrease urine supersaturation and stone risk factors, thus preventing recurrence [3,21]. Nevertheless, as shown in Table 4, when considering osmole intake assessed by eOsm value, a threshold of <900 mosm/Day increases by 19.2 and 6.5 folds a relative risk for sodium and protein intake within required targets (below 8 g/day of NaCl and 80 g/day of proteins). Therefore, eUosm appears as an accurate surrogate marker for osmolality in 24 h urine collection, providing also valuable information about the patient’s diet and water intake. We presume that in patients with altered renal concentrating ability, the association would be improved, as the concentration of urea (which is dependent on the renal gradient) would be less variable in the urine. Of note, fasting urine osmolality or conductivity provides no relevant information about diet except for insufficient diuresis with a 4.8- and 4.2-fold increased risk when mUosm or eOsm > 700 mosm/kg (*p* = 0.02 and 0.005, respectively).

## 6. Interest and Limitation of the Study

This retrospective study is cross-sectional and, thus, cannot assess the effectiveness of urine conductivity on stone recurrence, a goal that requires further studies. We speculate that urine conductivity could be a useful tool for stone risk factor screening and monitoring, especially in a primary care setting using 24 h urine collections, when urine biochemistry is difficult to perform. Whether our results could be extended to other populations remains to be tested. Among the potential biases, 24 h urine collection may indeed be > or <24 h in some patients, thus under- or overestimating dietary intake. However, as shown in Table 1, the fact that urine osmolality concentration was lower in the low-risk group despite an increased osmolality output (and 24 h urine creatinine) supports the view that low-risk patients “drink and eat more” but “drink even more…” rather than a 24 h urine collection bias. According to this view, stone risk factors such as urine calcium, phosphate, or urate concentrations are decreased in low-risk patients, as shown in Table S1 (supplementary data).

According to our data and following recommendations concerning measured urine osmolality, the renal stone patient monitoring target would be a 24 h urine eUosm value around 600–700 mosm/kg/day [4] and <350 mosm/kg in concentration, as this threshold increased the likelihood to meet all targets (namely diuresis > 2 L/day, sodium and protein intake below 9 g/day and 80 g/day, respectively) by 15.7-fold (*p* < 0.0001). Further prospective studies are nevertheless required to confirm the relevance of these targets.

This study has clinical and public health implications. From a clinical perspective, the measurement of urinary conductivity in 24 h urine can be used as a simple and reliable tool for estimating urinary osmolality, with a desired target below 350 mosm/kg in renal stone patients follow-up. In primary care, it would allow for more accessible outpatient monitoring, reducing the need for more costly or technical tests. In resource-limited countries, the predictive value of urinary conductivity could be a useful bedside, non-expensive, first-line tool, reducing the need for urine osmolarity measurement requiring a more expensive device and lab facilities. Indeed, conductivity meters are available in garden shops and stores for swimming pool gear at low cost (and also online…). Feasibility in routine practice seems excellent, no more difficult than a urine lab sticks reader or portable pH meter device.

## 7. Conclusions

Measuring urine conductivity instead of osmolarity determination appears as a non-expensive and reliable tool for screening and monitoring diet related renal stone patients in a primary care setting.

## Figures and Tables

**Table 1:** Comparison of demographic and biological parameters within 24 h urine collection and fasting urine sample in our renal stone population according to a diuresis < or >2 L/day.

	Whole Population	Diuresis < 2 L/Day	Diuresis > 2 L/Day	*p* Value
**Patients (n)**	n = 113	n = 52	n = 61	
**Female Gender (%)**	47%	51%	40%	NS
**24-h urine**				
**Diuresis (L/Day)**	1.95[1.5–2.5]	1.5[1.2–1.8]	2.6[2.3–2.8]	**<0.0001**
**Na (mmol/Day)**	130[85–165]	115[79–147]	142[103–186]	**0.01**
**K (mmol/Day)**	51[38–62]	41[35–55]	57[49–66]	**<0.0001**
**Urea (mmol/Day)**	318[248–386]	266[223–347]	374[301–473]	**<0.0001**
**Ca (mmol/Day)**	4.8[2.6–7.2]	4.7[1.8–7.1]	5.2[3.0–8.0]	0.22
**PO4 (mmol/Day)**	22[16–29]	22[15–26]	24[17–31]	0.08
**Urates (mmol/Day)**	3.4[2.6–4.2]	3.3[2.4–4.0]	3.7[2.7–4.8]	0.09
**Mg (mmol/Day)**	3.8[3.0–5.2]	3.5[2.9–5.0]	3.9[3.2–5.3]	0.53
**Oxalate (mmol/Day)**	0.34[0.22–0.49]	0.25[0.20–0.45]	0.40[0.29–0.52]	**0.01**
**Citrate (mmol/Day)**	2.3[1.5–3.3]	2.0[1.3–3.4]	2.5[1.8–3.2]	0.49
**Creatinine (mmol/Day)**	10.9[8.4–15.0]	9.9[8.0–14.2]	13.0[8.9–16.7]	**0.03**
**U pH**	6.3[5.7–6.8]	6.1[5.6–6.7]	6.5[6.0–7.0]	**0.009**
**U osm (mosm/L)**	396[287–498]	475[379–593]	331[253–427]	**<0.0001**
**Conductivity (mS/cm)**	8386[6365–11,785]	9092[7120–11,067]	6846[4892–8242]	**<0.0001**
**Fasting Urine**				
**U osm (mosm/L)**	526[407–680]	508[411–746]	580[411–642]	0.57
**Conductivity (mS/cm)**	10,331[7988–13,020]	11,209[8117–13,965]	9621[7119–12,041]	**0.04**

**Table 2:** Association between measured conductivity (mS/cm) or osmolality (mOsm/kg) and several urine parameters.

	Conductivity (mS/cm)		Osmolality (mosm/kg)	
	Rho	*p* value	Rho	*p* value
**24-h urine**				
**Diuresis**	−0.61	0.0002	−0.65	0.0002
**UNa (mmol/L)**	0.89	<0.0001	0.91	<0.0001
**UK (mmol/L)**	0.63	<0.0001	0.73	<0.0001
**U Urea (mmol/L)**	0.78	<0.0001	0.85	<0.0001
**U Ca (mmol/L)**	0.52	0.001	0.53	0.03
**U PO4 (mmol/L)**	0.54	0.0005	0.67	0.0001
**U creatinine**	0.81	<0.0001	0.87	<0.0001
**U pH**	−0.46	0.004	−0.46	0.007
**U osm (mosm/L)**	0.95	<0.0001	–	–
**Conductivity**	–	–	0.95	<0.0001

**Table 3:** Conversion table between urine measured conductivity and eUosm according to the following equation (eUosm = −41,656 +0.057 conductivity, r^2^= 0.93).

Urine Conductivity (µS/cm)	Urine Osmolality (mosm/kg)
1500	44
2000	72
2500	101
3000	129
3500	158
4000	186
4500	215
5000	243
5500	272
6000	300
6500	329
7000	357
7500	386
8000	414
8500	443
9000	471
9500	500
10,000	528
10,500	557
11,000	585
11,500	614
12,000	642
12,500	671
13,000	699
13,500	728
14,000	756
14,500	785
15,000	813
15,500	842
16,000	870
16,500	899
17,000	927
17,500	956
18,000	984
18,500	1013

**Table 4:** Analysis of risk factors associated with 24 h eUosm threshold < 900 mOsm/day (**A**) and 24 h eUosm threshold < 350 mOsm/kg (**B**) using univariate and multivariate stepwise logistic regression.

**Table 4A**	**Univariate**		**Multivariate**	
	**OR**	***p* value**	**OR**	***p* value**
**Diuresis > 2 L/day**	0.17[0.1–0.4]	<0.0001	0.20[0.06–0.73]	0.01
**U Na < 150 mmol/Day**	21.5[7.0–65.9]	<0.0001	19.2[5.1–72.2]	<0.0001
**U urea < 400 mmol/Day**	18.9[5–70]	<0.0001	6.4[1.4–28.8]	0.01
**No Hypercalciuria**	4.1[1.5–11.4]	0.006	–	–
**No Hyperoxaluria**	4.1[1.2–14.4]	0.02	–	–
**Table 4B**	**Univariate**		**Multivariate**	
	**OR**	***p* value**	**OR**	***p* value**
**Diuresis > 2 L/day**	3.6[1.5–8.6]	0.005	15.2[4.2–55.4]	<0.0001
**U Na < 150 mmol/Day**	9.2[2.5–32.9]	0.0007	16.5[3.6–75.7]	0.0003
**U urea < 400 mmol/Day**	3.5[1.1–11.2]	0.03	7.3[1.5–35.0]	0.01
**No Hypercalciuria**	3.0[0.9–9.7]	0.07	–	–
**No Hyperoxaluria**	2.1[0.5–8.8]	0.29	–	–

## Data Availability

The raw data supporting the conclusions of this article will be made available by the authors on request.

## Supplementary Materials

The following supporting information can be downloaded at: https://www.mdpi.com/article/10.3390/jcm14196898/s1, Figure S1: Association between daily diuresis and measured 24 h urine osmolality expressed in concentration (A) or daily output (B) or measured 24 h urine conductivity expressed in concentration (C) or daily output (D); Table S1: Comparison of urine parameters in our population according to low versus high-risk group (respectively, >2 or <2 L/day). As shown urine calcium, phosphate or urate concentrations are decreased due to urine dilution ruling out reasonably a bias due to incomplete urine collection in the high-risk group.

## Abbreviations

BRI	Bonn Risk Index
RSS	Relative Supersaturation
RRFA	Risk Robertson Risk Factor Algorithms
