# Supplementary material for: Estimated Osmolality by Measured Conductivity in 24 h Urine Renal Stone Patients: A Useful Tool for Monitoring Dietary Sodium and Protein Excess
Source: J Clin Med. 2025 Sep 29;14(19):6898. doi: 10.3390/jcm14196898 (PMC12524349; doi:10.3390/jcm14196898)
Supplement: Supplementary file 1 [file jcm-14-06898-s001.zip › jcm-3776951-supplementary.pptx]

## Slide 1
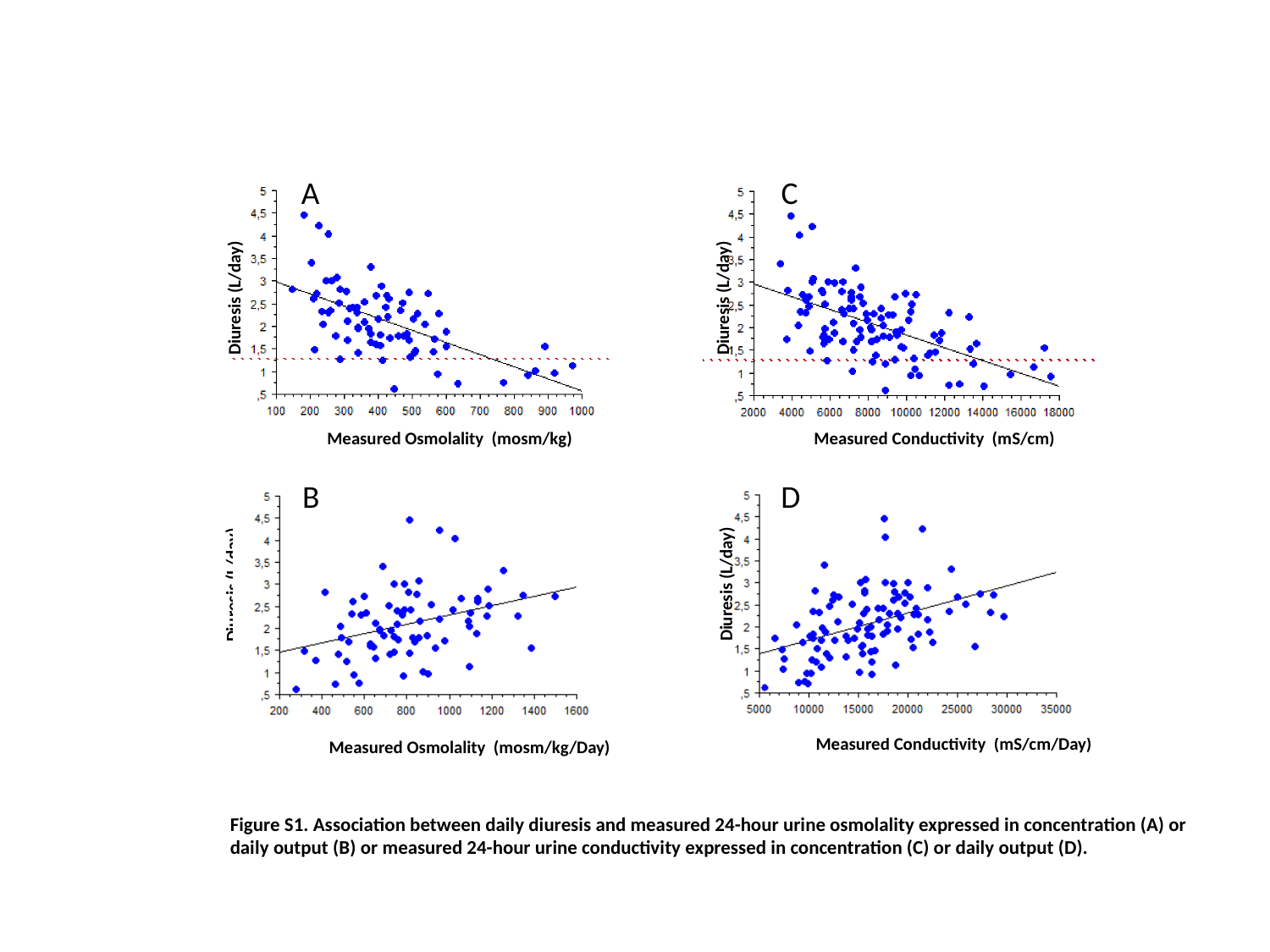

A
C
Diuresis (L/day)
Diuresis (L/day)
Measured Conductivity (mS/cm)
Measured Osmolality (mosm/kg)
B
D
Diuresis (L/day)
Diuresis (L/day)
Measured Conductivity (mS/cm/Day)
Measured Osmolality (mosm/kg/Day)
Figure S1. Association between daily diuresis and measured 24-hour urine osmolality expressed in concentration (A) or daily output (B) or measured 24-hour urine conductivity expressed in concentration (C) or daily output (D).

## Slide 2
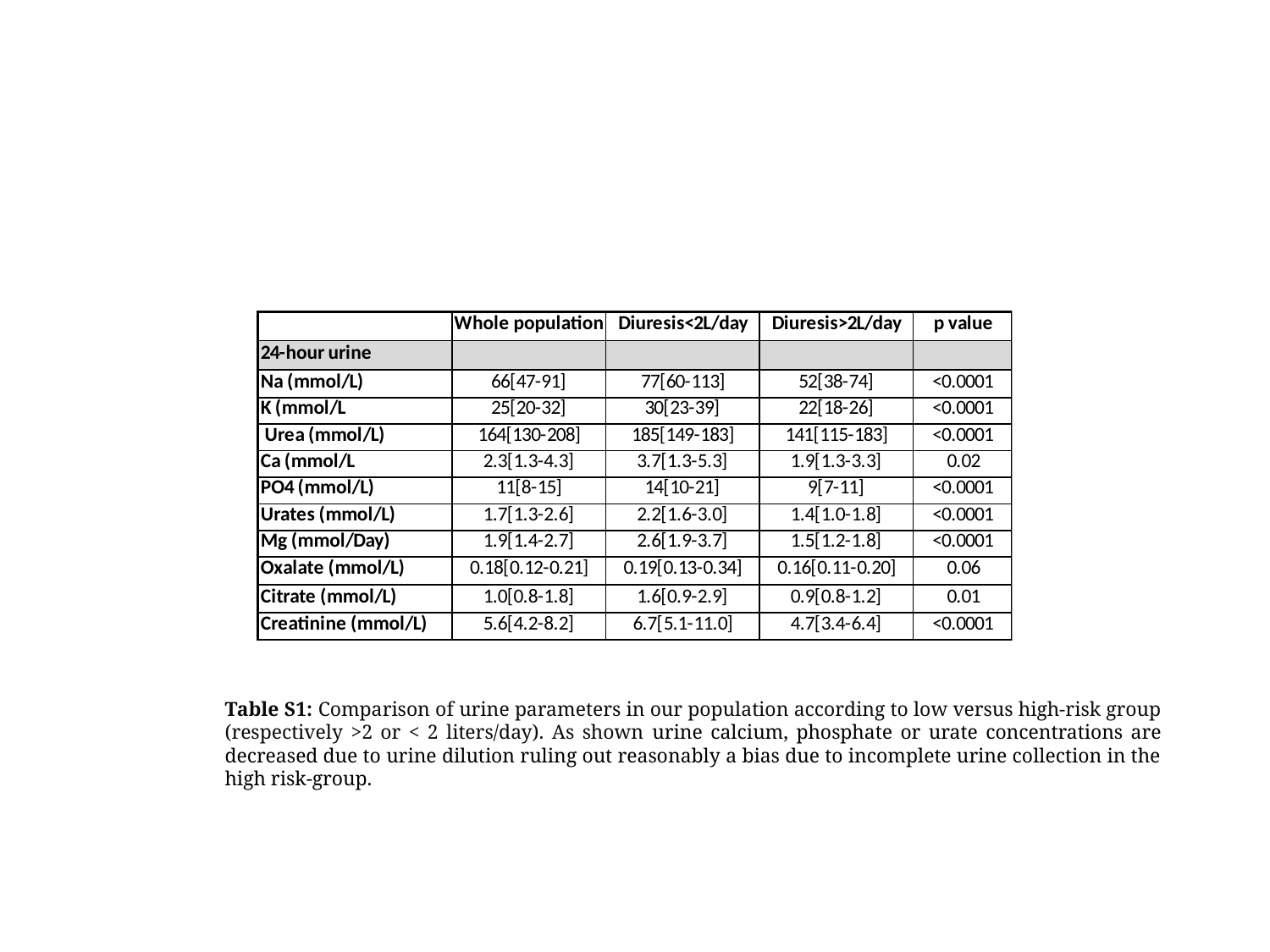

Table S1: Comparison of urine parameters in our population according to low versus high-risk group (respectively >2 or < 2 liters/day). As shown urine calcium, phosphate or urate concentrations are decreased due to urine dilution ruling out reasonably a bias due to incomplete urine collection in the high risk-group.
